# High varus stem alignment in short-stem total hip arthroplasty: a risk for reconstruction of femoro-acetabular offset, leg length discrepancy and stem undersizing?

**DOI:** 10.1007/s00402-021-04176-5

**Published:** 2021-09-23

**Authors:** Matthias Luger, Julian Stiftinger, Jakob Allerstorfer, Rainer Hochgatterer, Tobias Gotterbarm, Lorenz Pisecky

**Affiliations:** 1 Department for Orthopedics and Traumatology, Kepler University Hospital GmbH, Krankenhausstrasse 9, 4020 Linz, Austria; 2 Johannes Kepler University Linz, Altenberger Strasse 69, 4040 Linz, Austria; 3 Kepler University Hospital Linz, Krankenhausstrasse 9, 4020 Linz, Austria

**Keywords:** Short stem, Total hip arthroplasty, Varus, Femoral offset, Hip offset, Leg length

## Abstract

**Purpose:**

Short stems are increasingly used in in total hip arthroplasty (THA) because of advantages in bone and soft tissue preservation and reconstruction of hip geometry. Short stems can be inserted in a more varus position compared to conventional straight stems. This poses the risk of final varus misplacement of the femoral component, which is not intended in all femoral short stems.

**Methods:**

We wanted to evaluate the effect of a high varus stem positioning in MIS THA on hip offset, leg length and femoral canal fill index. A series of 1052 consecutive THAs with a singular cementless femoral short stem and press-fit cup was retrospectively screened for inclusion. One hundred six patients with unilateral THA and a contralateral healthy hip met the inclusion criteria. Measurements were carried out on preoperative and 3 months anterior–posterior postoperative radiographs. Patients were divided into Group A (varus stem alignment ≤ 3°) and Group B (varus stem alignment > 3°).

**Results:**

Hip offset (HO) increased significantly in Group B by 4 mm (*p *= 0.013). No influence on leg length difference was detected in both groups. Preoperative CCD angle was significantly lower in Group B (*p *< 0.001). Canal Fill Indices (CFI) were significantly lower in Group B (CFI I: *p *< 0.001; CFI II *p *= 0.003; CF III *p *= 0.002).

**Conclusion:**

High varus stem alignment > 3° leads to a statistically significant but minor increase in HO and poses the risk of stem undersizing. A preoperatively low genuine CCD angle pose a risk for varus stem positioning.

## Introduction

Usage of short-stem total hip arthroplasty (THA) systems has rapidly increased within the last years because of different theoretical advantages [1–3]. According to the literature, short stems are superior in preservation of proximal bone stock compared to standard straight stems [4–6]. Conventional stems with diaphyseal or metadiaphyseal anchorage may lead to unwanted stress shielding, enlargement of the effective joint space, aseptic loosening and potential bone loss, that may not retain enough intact bone for revision surgery compared to short stems [7, 8].

Another advantage of femoral short stems is the superior reconstruction of genuine hip geometry [1, 9]. Conventional straight stems show excellent long-term outcomes [10], but have the disadvantage of limited ability to restore the femoral offset (FO) due to their straight stem design [11]. Besides leg length (LL), FO influences the postoperative outcome, dislocation rate, wear and revision rate. Restoration of the native FO increases postoperative range of motion, abductor muscle function and decreases polyethylene wear [11–13]. Several studies even suggest a beneficial effect of an increased FO on abductor muscle force and joint reaction [14, 15]. Given these findings, the correct reconstruction of femoro-acetabular offset and LL has a high clinical relevance.

Apart from bone preservation and superior reconstruction of hip geometry, femoral short stems can be more soft tissue conserving, when minimally invasive approaches are performed [2, 16, 17]. A short curved stem can be inserted initially in a more varus position following a c-shaped path [2]. However, the final position of a short stem depends on its stem design, fixation and the level of osteotomy. The variety of femoral short stems is very high and a uniform classification is not available. Khanuja et al. [5] classified femoral short stems according to the fixation principle. Jerosch et al. [18] classified femoral short stems according to the level of femoral neck resection: femoral neck retaining (NR), femoral neck sparing (NS), and femoral neck harming (NH) short stems. Compared to neck retaining or neck sparing short stems, neck harming femoral short stems do not retain the femoral neck and consequently do not rely on the femoral neck in reconstruction of femoro-acetabular offset. The reconstruction of femoral offset heavily depends on the correct offset option of the used femoral short stem. Therefore, the aim in neck harming short-stem arthroplasty is an implantation oriented in line with the diaphysis [2].

Correct reconstruction of femoro-acetabular offset and leg length are clinically important factors [1, 19, 20]. Apart from medialization of the acetabular offset, increase of femoral offset by varus placement of the femoral stem can potentially influence the clinical and functional outcome after THA. Therefore, the present study was conducted to examine the influence on reconstruction of femoro-acetabular offset, leg length difference and femoral canal fill of a neck-harming femoral short stem in high varus position.

## Methods

### Study cohort

**Fig. 1:**
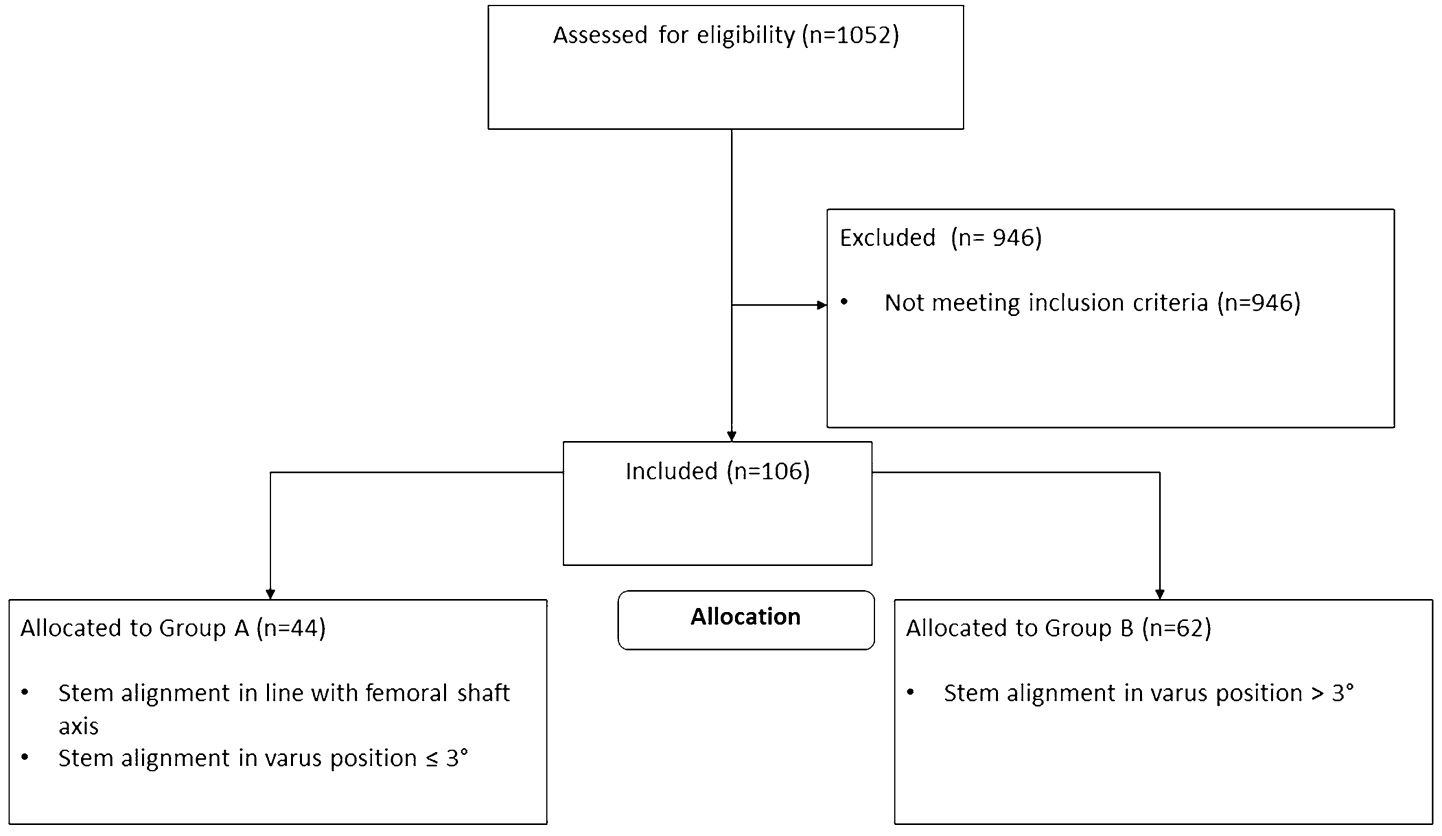
Consort diagram

This retrospective radiological comparative study includes patients of a consecutive series of THAs with the same cementless curved short stem (Fitmore^®^ stem, ZimmerBiomet, Warsaw, IN, USA) and bihemispherical press-fit acetabular cup (Allofit^®^/-S, ZimmerBiomet, Warsaw, IN, USA) performed via a minimally invasive supine anterolateral approach. A consecutive series of 1052 hips in 982 patients with index surgery between 2014 and 2019 were screened for inclusion and the medical records until 90 days postoperative were evaluated. Preoperative X-rays (both hips in comparison, anterior–posterior view, standing upright) were screened for unilateral THA. Exclusion criteria were defined as a contralateral hip disease (Kellgren Lawrence > grade 1) [21], a history of prior hip surgery, previous trauma, postoperative complication, reoperation or revision for any reason as well as missing pre- or postoperative radiographs. Diagnoses for inclusion were primary osteoarthritis, avascular necrosis of the femoral head or mild dysplasia of the hip (Crowe I) [22]. In total, 106 patients met the inclusion criteria. These patients were then divided into two groups according to the postoperative stem alignment (see Fig. 1, consort diagram). Stem alignment was measured as the difference in degrees between the anatomic femoral shaft and the vertical stem axis [23]. Groups were divided into group A with a stem alignment in line with the femoral shaft axis or with a varus stem alignment of ≤ 3° and into group B with a varus stem alignment of > 3°. In total, 44 patients could be allocated to group A and 62 patients could be allocated to group B. There is a high heterogeneity for cut-off values for the definition of varus stem alignment used in the literature with studies defining varus stem alignment > 1° [24, 25], > 3° [26] or > 5° [27]. However, as the used curved short stem in presented study often generally provokes a higher varus stem alignment due to its c-shaped insertion while broaching [2, 28], a higher value for diving the groups had to be defined. A stem alignment > 1° was, therefore, considered to be too low and a varus stem alignment > 5° was rare in the study group. In order to reach comparable group sizes 3° of varus stem alignment was chosen in this study. Radiographic measurements were performed on pre- and 3 months postoperative low-centered anteroposterior (AP) radiographs of the pelvis in both groups. Preoperative age at operation, gender, body mass index (BMI) and laterality were recorded. The patient demographics are shown in Table 1.

**Table 1:** Patient demographics, (mean and SD) and testing

Variable	Group A	Group B	*p* value
Number of Hips	44	62	–
Side (L:R)	20:24	29:33	1.000
Gender (F:M)	29:15	36:26	0.428
Age (years)	55.5 ± 10.9 (32.6–77.1)	58.2 ± 11.3 (27–76.8)	0.294
BMI at surgery	28.3 ± 6 (18.8–47.3)	27.1 ± 4.1 (17.9–36.1)	0.415

*SD* standard deviation, *F* female, *M* male, *L* left, *R* right, *BMI* Body Mass Index (kg/m^2^)

The study was approved by the institutional review board (EK-No.: 1239/2019). Due to the retrospective study design with evaluation of pre-existing medical records an informed consent was not required. All procedures performed in studies involving human participants were in accordance with the ethical standards of the institutional and/or national research committee and with the 1964 Helsinki declaration and its later amendments or comparable ethical standards.

### Surgical technique and treatment protocol

Surgical procedures were carried out at the author’s institution by surgeons with different levels of experience including 11 consultants and 7 residents. All consultants perform more than 50, all senior consultants more than 100 arthroplasties per year. Resident surgeries were done under the guidance of a consultant. In all cases, a minimally invasive anterolateral Watson-Jones approach in supine position on a standard operating table under laminar air-flow was performed. Extremity preparation was performed with threefold antiseptic scrub with alcohol disinfectant. Draping with a sterile adhesive surgical iodine film was used. The skin incision was centered over the greater trochanter. An incision at the border between the Tensor fasciae latae and the Tractus iliotibialis was performed. Then, the Watson-Jones interval between Tensor fasciae latae and Gluteus medius was bluntly dissected. A capsulectomy was performed in every case. Fluoroscopy was not routinely used. The standardized peri- and postoperative protocol was identical in all cases, including single-shot antibiotics (Cefuroxime 1.5 g i.v. directly preoperative), weight-bearing as tolerated from the first postoperative day on, Indomethacin 75 mg daily for the prevention of heterotopic ossification on days 1–4 postoperatively and 40 mg low-molecular weight heparin or Rivaroxaban 10 mg for 28 days postoperatively as venous thromboembolic event prophylaxis.

In all patients a cementless, curved short stem (Fitmore^®^ stem, ZimmerBiomet, Warsaw, IN, USA) Fitmore^®^ hip stem is a titanium alloy stem (Ti Al6V4) that has a porolock Ti-VPS coating in the proximal part to enhance bone ingrowth and is available in four different neck angle options (127°, 129°, 137°, 140°) [2]. A cementless titanium press-fit cup with or without screws (Allofit^®^/-S, ZimmerBiomet, Warsaw, IN, USA) was used in all patients. Digital templating was performed prior to surgery in all cases using mediCAD^®^ version 5.1 (Hectec GmbH, Altdorf, Germany).

### Radiographic evaluation

**Fig. 2:**
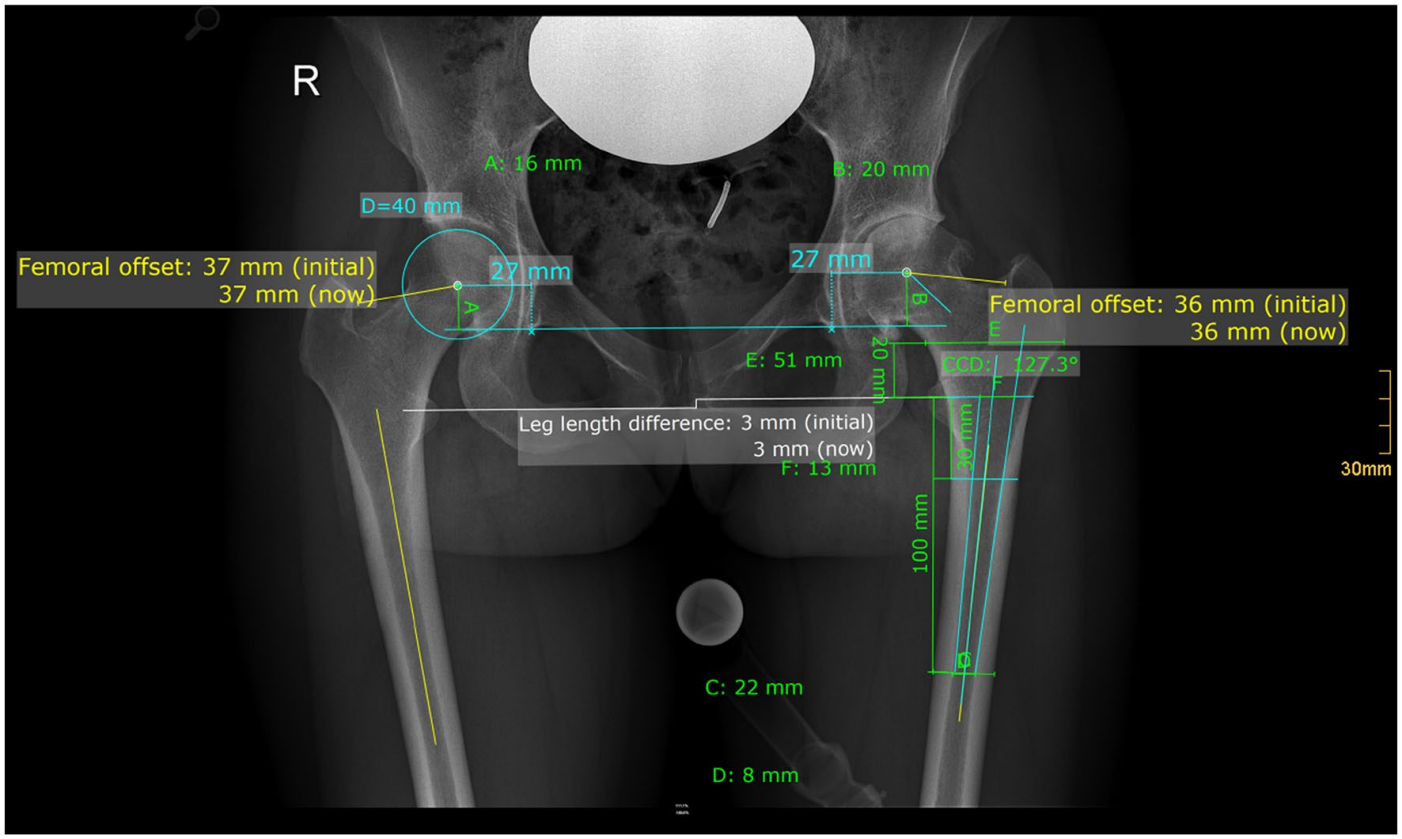
Preoperative measurements: both sides: femoral offset (FO), acetabular offset (AO), vertical position of the center of rotation (COR), leg length difference (LLD); affected hip: Centrum-Collum-Diaphyseal Angle (CCD angle), Cortical Index (CI), Canal Flare Index, Canal to Calcar ratio

Radiographic measurement was performed on preoperative and 3 months postoperative digital low-centered AP radiographs of the pelvis [29]. Measurements were conducted independently by two reviewers (M.L., J.S.), who were not involved in index surgery. Radiographs were taken with the patient in standing position and with both legs in 15° internal rotation and the central beam was directed on the symphysis pubis [20]. To achieve an accurate measurement of the hip anatomy, a double coordinate system was applied on both the preoperative and the postoperative images [1, 30]. Radiographic analysis was done using MediCAD^®^ Software V5.1 (Hectec GmbH, Altdorf, Germany). The hip center of rotation (COR) was defined using a circle tool determining the diameter of the femoral head and its center [31]. The femoral offset (FO) was determined as the perpendicular distance between the COR and the proximal femoral shaft axis (FSA) [29, 31]. Acetabular offset (AO) was measured as the perpendicular distance between the COR and line T, with T being the perpendicular line on the transteardrop line (TT) through the ipsilateral teardrop figure [29]. Hip offset (HO) was calculated as the sum of FO and AO [29]. The vertical position of the COR was measured as the perpendicular distance to line TT [32]. Radiographic leg length difference (LLD) was measured as the perpendicular distance between line TT and the middle of the lesser trochanter (LT) [20]. Centrum-Collum-Diaphyseal (CCD) angle was determined according to M. E. Müller on the affected hip [33]. To characterize the anatomical shape of the proximal femur and the thickness of cortical bone, the canal to calcar isthmus ratio and the cortical index (CI) according to Dorr et al. [34] were determined. A high CI indicates a thick cortical bone [34]. Additionally the canal flare according to Noble et al. [35] was determined. The stem alignment was measured as the difference in degrees between the anatomic femoral shaft axis and the vertical stem axis [23]. The canal fill index (CFI) was determined to evaluate the metaphyseal/diaphyseal filling of the femoral canal by the cementless stem implant on 3 different heights (CFI I: at the level of the LT, CFI II: 1 cm below the LT, CFI III: 3 cm below the LT). On each height, the horizontal diameter of the stem implant was measured and divided by the endosteal medullary canal diameter, multiplied by 100 to achieve the relative percentage [32]. Cup inclination was defined as the angle between the TT line and the line connecting the most superior and inferior aspect of the cup. Cup anteversion was measured and calculated according to the formula by Lewinnek et al. [36], as recently validated by computer tomography based data [37]. On preoperative x-rays FO, AO, HO and LLD and vertical position of the COR were measured bilaterally, while CCD angle, CI, Canal Flare Index and Canal to Calcar Ratio were measured unilaterally on the affected hip. Complete preoperative measurements are also shown in Fig. 2.

**Fig. 3:**
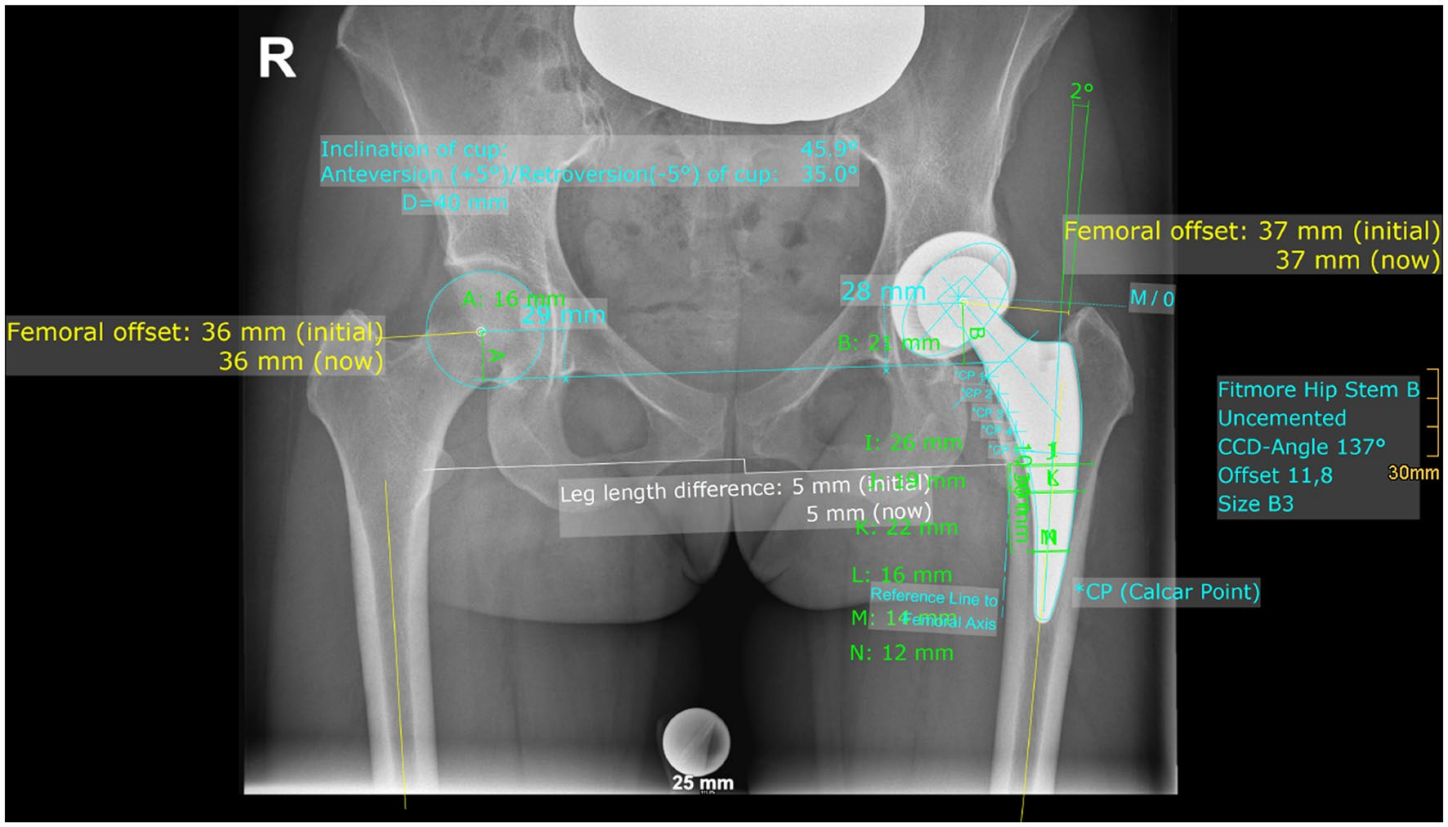
Both sides: femoral offset (FO), acetabular offset (AO), vertical position of the center of rotation (COR), leg length difference (LLD); affected side: stem alignment, canal fill index I, II and III, cup inclination, cup anteversion

On postoperative X-rays FO, AO, HO, LLD and vertical position of the COR were measured bilaterally, while cup inclination, cup anteversion, stem alignment, CFI, CFII and CFIII were measured unilaterally on the operated hip. Complete postoperative measurements are also shown in Fig. 3.

Intra- and interobserver reliabilities were calculated for 15 randomly selected cases for each group [20, 38]. Intra-class-correlation coefficients (ICC) were used with a two-way random effects model for absolute agreement. Repeated measurements for intraobserver reliability were performed at day 1 and day 14 in a blinded fashion.

### Statistics

Testing for statistical significance in postoperative stem alignment was performed to confirm a significant difference in both groups prior to further statistical analysis. The division into described groups was confirmed statistically (*p *< 0.001) as shown in Table 3.

Descriptive statistical analysis was conducted for Age, gender, body mass index (BMI) and laterality. A Shapiro–Wilk Test was performed for testing for normal distribution. As not all variables were normally distributed non-parametric testing was performed. For patient demographics, a Fisher’s exact test was performed on categorical variables (gender and laterality) in order to evaluate any association between independent variables and the likelihood of a fracture. Post hoc calculations with Bonferroni correction were not carried because of missing statistical significance. A Wilcoxon Mann–Whitney *U* Test was performed on continuous variables (age and BMI). For statistical analysis of pre- and postoperative radiographic measurements a non-parametric Wilcoxon Mann–Whitney *U* Test was performed. A Pearson’s correlation was performed between the variables preoperative CCD angle and postoperative stem alignment. Statistical analysis was calculated with SPSS version 26 (IBM SPSS statistics, Chicago, IL, USA). A *p* value < 0.05 was considered as statistically significant.

## Results

The interobserver and intraobserver correlation coefficient for radiographic measurements showed satisfying results [range 0.961 (95% CI 0.853–0.989) to 0.998 (95% CI 0.986–0.999)]. Average age at operation, gender, BMI and laterality were evenly distributed and did not show any statistical significance. Detailed demographic data for both groups is given in Table 1. Preoperative radiological differences between affected and contralateral healthy hip are shown in Table 2. Difference in HO, AO, FO, LLD and vertical position of the COR did not show any statistical difference for both groups. Preoperative CCD angle was significantly lower in Group B (*p *< 0.001). Pearson’s *r* was − 0.474 (*p *< 0.001), showing a significant correlation between low preoperative CCD angle and high postoperative varus stem alignment. Anatomical shape of the affected side did not show any significance in testing the Cortical Index, Canal Flare Index and Canal to Calcar ratio.

**Table 2:** Preoperative radiographic measurements

Preoperative discrepancy between arthritic and healthy hip	Group A	Group B	P Value
Hip offset (mm)	1.5 ± 4	0.8 ± 4.1	0.399
Acetabular offset (mm)	0.7 ± 2	0.9 ± 2.8	0.838
Femoral offset (mm)	2.3 ± 3.9	1.7 ± 4.3	0.598
Leg length difference (mm)	− 4.2 ± 4.3	− 3.5 ± 5.2	0.42
Vertical position of the COR (mm)	3 ± 4.2	2 ± 3.3	0.139
CCD angle (°)	135 ± 6	128.9 ± 5.7	< 0.001
Cortical index	0.59 ± 0.05	0.61 ± 0.05	0.05
Canal flare index	4.65 ± 0.75	4.73 ± 0.76	0.670
Canal to calcar ratio	0.61 ± 0.08	0.62 ± 0.1	0.621

All values are expressed as mean ± standard deviation

*COR* center of rotation, *CCD angle* centrum-collum-diaphyseal angle

Table 3 shows the postoperative measurements in detail. Postoperatively FO increased significantly in both groups (Group A: 4.8 mm; Group B: 8.1 mm; *p *< 0.001) compared to the contralateral healthy hip, while AO was decreased significantly in both groups (Group A: − 3.3 mm; Group B: − 4.1 mm; *p *< 0.001). HO only increased significantly in Group B by 4 mm (*p *= 0.013), while Group A did show an increase of 1.4 mm without any statistical significance compared to the healthy contralateral side (*p *= 0.366). The vertical position of the COR was significantly placed superiorly in both groups (Group A: 4.9 mm; Group B: 4.7 mm; *p *< 0.001). Leg length difference (LLD) was decreased by 2.9 mm in group A and 4.2 mm in Group B with a postoperative LLD of − 1.3 mm for Group A and 0.7 mm for Group B. Testing for significance in postoperative LLD did not show a significance (*p *= 0.058). All Canal Fill Indices were significantly lower in Group B (CFI I: *p *< 0.001; CFI II *p *= 0.003; CF III *p *= 0.002). Placement of acetabular cup did not show any difference in inclination and anteversion between both groups.

**Table 3:** Postoperative radiographic measurements

Variable	Group A		Group B	
	THA	Healthy hip	THA	Healthy hip
Hip offset (mm)	69.8 ± 5.8	68.4 ± 6.2	79.1 ± 8.6	75.1 ± 7.6
*p* value	0.366		0.013	
Acetabular offset (mm)	29.3 ± 3.7	32.6 ± 3.7	28.8 ± 3.4	32.9 ± 4.5
*p* value	< 0.001		< 0.001	
Femoral offset (mm)	40.5 ± 5.4	35.7 ± 4.6	50.2 ± 7.5	42.1 ± 5.2
*p* value	< 0.001		< 0.001	
Vertical position of the COR (mm)	18.8 ± 4.1	13.9 ± 3.4	19.3 ± 3.7	14.6 ± 3.8
*p* value	< 0.001		< 0.001	
Leg length difference^a^ (mm)	− 1.3 ± 5.5		0.7 ± 4.7	
*p* value	0.058			
Stem alignment (°)	1.8 ± 1.1		6.7 ± 2.3	
*p* value	< 0.001			
Canal fill index I	80.88 ± 5.99		76.14 ± 5.87	
*p* value	< 0.001			
Canal fill index II	83.21 ± 6.28		80.74 ± 6.8	
*p* value	0.003			
Canal fill index III	86.81 ± 6.74		81.01 ± 9.32	
*p* value	0.002			
Cup inclination (°)	44.8 ± 6.44		43.27 ± 6.08	
*p* value	0.428			
Cup anteversion (°)	29.09 ± 5.63		27.43 ± 5.96	
*p* value	0.131			

All values are expressed as mean ± standard deviation

^a^Between healthy and operated hip

## Discussion

Accurate reconstruction of hip geometry in THA is essential and has influence on clinical outcome, dislocation risk, range of motion, impingement, abductor muscle strength, and polyethylene wear [39–41]. To our knowledge, no study has yet addressed the influence of a high varus stem alignment in neck-harming short-stem THA on femoro-acetabular offset and leg length.

The impact of offset reconstruction on the clinical outcome has been extensively examined. Innmann et al. [19] reported the best improvement in clinical outcome with a combination of complete to slightly increased HO (± 5 mm) reconstruction and a marginal LLD in short-stem THA with Fitmore^®^ hip stem. Mahmood et al. [39] reported weaker hip abductor muscle strength in patients with a decrease in HO by more than 5 mm. Sariali et al. [41] reported comparable findings with altered gait with asymmetry between both hips, reduced range of motion, and a lower maximal swing speed on the operated side for patients with a minimum decrease in FO of 15%. Cassidy et al. [15] reported that a decrease in FO of more than 5 mm resulted in worse Western Ontario and McMaster Universities Arthritis Index (WOMAC) scores compared to patients with reconstructed or increased FO. Our results show an increase of HO by 1.4 mm in THA with ≤ 3° varus stem alignment and 4 mm in THA with high varus stem alignment > 3° compared to a healthy contralateral hip. Additionally, an increase in HO of ≥ 5 mm compared to the contralateral normal hip negatively effects polyethylene wear [40]. We report values in a range under an increase of 5 mm for both groups, which is suggested to be superior for clinical functional outcome [19] and for polyethylene wear [40]. High varus stem alignment leads to a significant increase in HO. A varus stem alignment > 3° in short-stem THA with Fitmore^®^ hip stem leads to a tolerable increase of 4 mm. A stem positioning in line with the femoral shaft axis or with a low varus position ≤ 3° leads to significantly better reconstruction of femoro-acetabular offset compared to a contralateral healthy hip. Therefore, high varus positioning is critical because of a loss of control of the increase in HO, especially in high offset variants such as Fitmore Typ B extended or Typ C (Typ B extended: CCD-Angle 129°; Typ C: 127°). A high varus stem alignment can be misleading intraoperatively. Offset option Typ B (CCD 137°) and offset option Typ B extended (CCD 129°) do not differ in stem size, but differ in CCD angle and offset. In case of instability in intraoperative trial reduction with offset option Typ B, Fitmore^®^ hip stem shows the advantage of changing to offset option Typ B extended without any need for further broaching because of identical stem size. However, if the trial rasp is positioned in a high varus position, the impact on hip offset with a higher offset option is difficult to evaluate intraoperatively and can be misleading. Therefore, the danger of disproportionate increase of HO should be considered. Our results show only mild impact on HO in high varus stem alignment. Final placement of Fitmore stem is intended in line with the diaphysis [2]. However, the effect of increase in HO is only minimal in high varus alignment.

A high varus stem alignment did not pose a risk for leg length difference. Adequate reconstruction of HO and LL is considered as clinically important in THA [19, 29]. However, the literature on leg length difference after THA and its clinical influence are inconsistent [19]. The consensus agreement recommends a LLD, that is kept to a minimum [39, 42]. We report a sufficient restoration of leg length with minimal average LLD in both groups. We therefore conclude, that LLD can be kept at a minimum independently from varus positioning of the femoral component in short-stem THA with the Fitmore^®^ hip stem.

Canal Fill Indices for the Fitmore^®^ hip stem are reported with CFI I of 85.6% ± 5.4, a CFI II of 90.4% ± 6.9 and a CFI II of 85.2% ± 11.1 [32]. We report lower Canal Fill Indices for both groups at all measurement points. Canal Fill Indices range from 80.88% to 86.81% in Group A and are significantly lower in high varus ranging from 76.14% to 81.01%. A Canal Fill Index < 80% is considered as undersized [43]. Because of a higher varus position, growth in size while broaching is limited. As a result, a lower Canal Fill is achieved with less contact against the endosteal cortex of the calcar and the medial and lateral metaphyseal/diaphyseal area. Therefore, an implantation in high varus alignment could pose a risk for primary stability and bony ingrowth. The Fitmore^®^ hip stem shows a high primary stability comparable to straight stem systems [44]. The survival rate of the Fitmore^®^ hip stem after 8.6 years is reported with 99.6% (95% CI 97.1–99.9%) for the endpoint of “stem revision due to aseptic loosening”. However, the long-term impact of a low Canal Fill Index because of a high varus placement is not fully known.

The reason for varus placement of femoral shafts is also discussed in literature. Less surgical exposure in minimally invasive approaches could lead to broaching in a more varus position [2]. Another reason for varus stem alignment could be preoperative CCD angle. Murphy et al. [45] demonstrated that low CCD-angles and Coxa vara deformity leads to varus implantation in cementless straight stem THA. In the presented study, a significant difference in genuine CCD angle was found (*p *< 0.001). The group with high varus stem alignment had a significantly lower genuine CCD angle. This was also demonstrated with a statistically significant correlation analysis. Therefore, we postulate, that a low preoperative genuine CCD angle could pose a risk factor for high varus stem placement in short-stem THA with Fitmore^®^ hip stem. Therefore, surgeons should pay attention to low CCD angles preoperatively.

Fluoroscopy was not used routinely in the included cases. Routine use of fluoroscopy would be a potential procedure to reduce stem malalignment, undersizing of the femoral implant and prevention of periprosthetic fractures. Studies investigating the accuracy of implant positioning mainly focus on cup positioning [46–49]. The effect on implant positioning with the use of fluoroscopy is mixed with studies showing improvements for cup positioning and leg length difference by the use of fluoroscopy [48, 49]. However, Bingham et al. [47] reported similar results without any statistical significance for leg length difference in THA with and without fluoroscopy. Additionally, there is no clear evidence for a significant improvement of clinical outcome by routine use of fluoroscopy.

Several limitations of the study have to be addressed. First, we tried to minimize a potential selection bias with very strict inclusion criteria. We present a consecutive cohort with over 1000 THAs, that was reviewed for inclusion. Only patients with a single implant design and approach were included. A homogenous study cohort was created by excluding patients with a contralateral hip disease (Kellgren Lawrence > grade 1). Both study groups were tested for differences in age at surgery, BMI, laterality and gender without any significance. Also, preoperative measurements were tested for statistically significant differences. Only CCD angle was significantly different between both groups. However, this was interpreted as a consequence of forming two groups based on postoperative stem alignment, rather than being a selection bias. Furthermore, we aimed to increase reliability of the measurements and results by restricting inclusion based on preoperative diagnosis. We excluded all forms of secondary osteoarthritis of the hip and development dysplasia of the hip Crowe grades II–IV. Prior surgery before THA was also excluded. However, mild hip dysplasia (lateral center–edge angle 20–25°), coxa profunda, and morphologic alterations related to cam- or pincer-type impingement were included, because these changes might be subtle and cannot be reliably identified in the present study cohort with end-stage disease. Therefore, we conclude, that the findings in the present study are applicable for primary osteoarthritis and care must be taken when applying our findings on secondary osteoarthritis or high grades of development dysplasia of the hip. Secondly, we address the fact of taking measurements on plain radiographs. FO is underestimated by approximately 13% on plain radiographs [50]. Additionally, radiographic measurement of leg length difference does not necessarily reflect clinical leg length difference [51]. However, our measurements are easily reproducible, applicable in daily routine and less invasive regarding radiation exposure. Furthermore, we postulate variances in inter- and intraobserver reliability in measuring clinical leg length difference. We acknowledge the restrictions of measurements on plain radiographs. But with implementing strict inclusion criteria and by using reproducible and well described landmarks for measuring, we postulate a sufficient reduction of these limitations. An additional limitation of this study is missing clinical outcome measures or patient reported outcome measures. However, the aim of this study was to evaluate impact of offset reconstruction, leg length difference and implant positioning and sizing depending on different stem alignments. Further research is needed to give a definitive verdict on the clinical impact of high varus stem alignment in short-stem THA. Furthermore, fluoroscopy was not routinely used. This might have been a key factor for higher varus stem alignment and stem undersizing. A routine use of fluoroscopy might have a significant impact in reduction of implant malpositioning.

## Conclusion

A varus stem alignment with more than 3° increases hip offset significantly compared to a contralateral healthy hip and leads to a significantly lower fill of the femoral canal with risk of undersizing in short-stem THA with a neck-harming femoral short stem. A preoperatively low genuine CCD angle pose a risk for varus stem positioning. Long-term effects on functional outcome and rate of aseptic loosening in high varus alignment has to be evaluated in further studies.
